# The Calpain Inhibitor MDL28170 Induces the Expression of Apoptotic Markers in *Leishmania amazonensis* Promastigotes

**DOI:** 10.1371/journal.pone.0087659

**Published:** 2014-01-31

**Authors:** Fernanda A. Marinho, Keyla C. S. Gonçalves, Simone S. C. Oliveira, Diego S. Gonçalves, Filipe P. Matteoli, Sergio H. Seabra, Ana Carolina S. Oliveira, Maria Bellio, Selma S. Oliveira, Thaïs Souto-Padrón, Claudia M. d'Avila-Levy, André L. S. Santos, Marta H. Branquinha

**Affiliations:** 1 Departamento de Microbiologia Geral, Instituto de Microbiologia Paulo de Góes (IMPG), Universidade Federal do Rio de Janeiro (UFRJ), Brasil; 2 Laboratório de Biologia Molecular e Doenças Endêmicas, Instituto Oswaldo Cruz (IOC), Fundação Oswaldo Cruz (FIOCRUZ), Rio de Janeiro, Brasil; 3 Laboratório de Tecnologia em Cultura de Células, Centro Universitário Estadual da Zona Oeste (UEZO), Rio de Janeiro, Brasil; 4 Laboratório de Imunologia Molecular, Instituto de Biofísica Carlos Chagas Filho, UFRJ, Rio de Janeiro, Brasil; 5 Departamento de Imunologia, IMPG, UFRJ, Brasil; 6 Instituto Nacional de Ciência e Tecnologia de Biologia Estrutural e Bioimagem (INBEB), UFRJ, Rio de Janeiro, Brasil; Instituto Oswaldo Cruz, Fiocruz, Brazil

## Abstract

**Background:**

Human cutaneous leishmaniasis is caused by distinct species, including *Leishmania amazonensis*. Treatment of cutaneous leishmaniasis is far from satisfactory due to increases in drug resistance and relapses, and toxicity of compounds to the host. As a consequence for this situation, the development of new leishmanicidal drugs and the search of new targets in the parasite biology are important goals.

**Methodology/Principal Findings:**

In this study, we investigated the mechanism of death pathway induced by the calpain inhibitor MDL28170 on *Leishmania amazonensis* promastigote forms. The combined use of different techniques was applied to contemplate this goal. MDL28170 treatment with IC_50_ (15 µM) and two times the IC_50_ doses induced loss of parasite viability, as verified by resazurin assay, as well as depolarization of the mitochondrial membrane, which was quantified by JC-1 staining. Scanning and transmission electron microscopic images revealed drastic alterations on the parasite morphology, some of them resembling apoptotic-like death, including cell shrinking, surface membrane blebs and altered chromatin condensation pattern. The lipid rearrangement of the plasma membrane was detected by Annexin-V labeling. The inhibitor also induced a significant increase in the proportion of cells in the sub-G_0_/G_1_ phase, as quantified by propidium iodide staining, as well as genomic DNA fragmentation, detected by TUNEL assay. In cells treated with MDL28170 at two times the IC_50_ dose, it was also possible to observe an oligonucleossomal DNA fragmentation by agarose gel electrophoresis.

**Conclusions/Significance:**

The data presented in the current study suggest that MDL28170 induces apoptotic marker expression in promastigotes of *L. amazonensis*. Altogether, the results described in the present work not only provide a rationale for further exploration of the mechanism of action of calpain inhibitors against trypanosomatids, but may also widen the investigation of the potential clinical utility of calpain inhibitors in the chemotherapy of leishmaniases.

## Introduction

According to the World Health Organization [1], cutaneous and visceral leishmaniases are included in the list of neglected tropical diseases, being considered a major global threat for health. Human cutaneous leishmaniasis is caused by distinct species in the Old and New World, including *Leishmania amazonensis* in the latter. This trypanosomatid is associated with all clinical forms of the disease, which includes localized, mucocutaneous and diffuse cutaneous leishmaniasis [2], and is commonly found in the Amazon region, encompassing many Latin American countries [3]. While localized cutaneous leishmaniasis has a tendency to spontaneously self-heal with resulting scars, no or incomplete treatment is associated with the subsequent development of mucocutaneous leishmaniasis [4]. Despite many options, treatment of cutaneous leishmaniasis is far from satisfactory due to increases in drug resistance and relapses, and toxicity of compounds to the host. In this context, the first-line drugs used for treatment of leishmaniasis are still pentavalent antimonial compounds, while amphotericin B and pentamidine are used as the second-line chemotherapy [5], [6]. As a consequence for this situation, the development of new leishmanicidal drugs and the search of new targets in the parasite biology are important goals.

The previous demonstration by our group that the viability of *L. amazonensis* is reduced by the dipeptidyl aldehyde calpain inhibitor MDL28170 (calpain inhibitor III, Z-Val-Phe-CHO) [7] encouraged the study to uncover the death mechanism promoted by this drug. This compound is a membrane-permeable cysteine peptidase inhibitor with a *K_i_* for calpain of 8 nM [8]. Originally developed for use against mammalian calpains, MDL28170 has been reported to have neuroprotective effects in numerous rodent neurotrauma models, including spinal cord injury, neonatal hypoxia-ischemia and focal cerebral ischemia, and also to reduce neuronal loss and improve locomotor functions in a mouse model of Parkinson's disease [9]. In all of these pathological processes, the role of deregulated calpain activation has been thoroughly demonstrated [10], [11]. A distinguishing feature of the proteolytic activity of calpains is their ability to confer limited cleavage of protein substrates, and as such calpain-mediated proteolysis represents a major pathway of post-translational modification that influences many aspects of cell physiology, including cell adhesion, migration, proliferation and apoptosis, among other functions [10], [12], [13].

Some of the effects of the calpain inhibitor MDL28170 were already determined by our group upon *L. amazonensis* and *T. cruzi* growth. Our results showed that this calpain inhibitor promoted cellular alterations and arrested the growth of the proliferative forms of both parasites in a dose-dependent manner [7], [14]. Previous works from our group also showed that MDL28170 acted against all the morphological stages found in *T. cruzi*, without displaying any relevant cytotoxic effect on mammalian host cells [14], [15]. The calpain inhibitor also arrested the *in vitro* epimastigote into trypomastigote differentiation and led to a significant reduction in the capacity of *T. cruzi* epimastigote adhesion to the insect guts of the insect vector *Rhodnius prolixus* in a dose-dependent manner [16]. In *L. amazonensis*, an 80-kDa calpain-like protein was identified on the cell surface of the parasite using an anti-calpain antibody developed against *D. melanogaster* calpain, and no cross-reactivity was found with mammalian calpains [7]. With these results in mind, we aimed to investigate in the present work the mechanism of cellular death promoted by this compound in *L. amazonensis* promastigotes. Through the combined use of different techniques, we found that MDL28170 induces the expression of apoptotic markers in these cells.

## Materials and Methods

### Parasite culture

Promastigotes of *Leishmania amazonensis* (MHOM/BR/75Josefa) were routinely cultured at 28°C in Schneider's medium supplemented with 10% heat-inactivated fetal bovine serum (FBS) and gentamicin (40 µg/mL).

### Multiplication inhibition assay

The effects of MDL28170 (purchased from Calbiochem, San Diego, CA) on promastigote forms of *L. amazonensis* were assessed by a method similar to that described previously [7]. Promastigotes were counted using a Neubauer chamber and resuspended in fresh medium to a final concentration of 10^6^ viable promastigotes per ml. The calpain inhibitor was added to the culture at final concentrations ranging from 10 µM to 30 µM (starting from a 5 mM solution in DMSO). Dilutions of DMSO corresponding to those used to prepare the drug solutions were assessed in parallel. After 72 h of incubation at 28°C, the number of late-log, viable motile promastigotes was quantified in a Neubauer chamber. This incubation period was chosen because a significant reduction in the growth rate was observed for late-log phase promastigotes in comparison to mid-log phase cells [7]. The 50% inhibitory concentration (IC_50_), i.e. the minimum drug concentration that caused a 50% reduction in survival/viability was determined by linear regression analysis by plotting the number of viable promastigotes versus log drug concentration using Origin Pro 7.5 computer software.

### Parasite viability assay

Resazurin dye/AlamarBlue® (7-Hydroxy-3H-phenoxazin-3-one 10-oxide) was employed for promastigote viability testing. Resazurin is a redox potential indicator that is converted to fluorescent and colorimetric resorufin dye by the metabolically active cells. Non-viable cells rapidly lose their metabolic capacity to reduce resazurin in the mitochondrion and, thus, do not produce fluorescent signals anymore [17]. Assays were performed in sterile 96-well plates using late log-phase promastigotes (5×10^5^ cells/well) in the absence (control) or in the presence of the IC_50_ or two times the IC_50_ doses of MDL28170. After 72 h of incubation at 28°C, 20 µL of resazurin [0.0125% (w/v) in PBS)] were added, and plates were incubated for a further 4 h at the same temperature. After incubation, cells were analyzed at a microplate reader (SpectraMax spectrofluorometer, Molecular Devices) using a pair of 590 nm and 544 nm as emission and excitation wavelengths, respectively. The viability was evaluated based on a comparison with untreated, control cells. Parasites were also treated with sodium azide (0.95 g/L) for 30 min in order to obtain non-viable cells to use as a positive control in the viability test.

### Estimation of mitochondrial transmembrane electric potential (Δψm)

The mitochondrial transmembrane electric potential (Δψm) of the control cells and MDL28170-treated (IC_50_ and two times the IC_50_) promastigotes was investigated using the JC-1 fluorochrome, which is a lipophilic cationic mitochondrial vital dye that becomes concentrated in the mitochondrion in response to Δψ*m*. The dye exists as a monomer at low concentrations, where the emission is at 530 nm (green fluorescence), but at higher concentrations it forms J-aggregates after accumulation in the mitochondrion, where the emission is at 590 nm (red fluorescence). Thus, the fluorescence of JC-1 is considered an indicator of an energized mitochondrial state, and it has been used to measure the Δψ*m* in *Leishmania* [18]. Control and MDL28170-treated promastigotes after 72 h of treatment were harvested, washed in PBS and added to a reaction medium containing 125 mM sucrose, 65 mM KCl, 10 mM HEPES/K^+^, pH 7.2, 2 mM Pi, 1 mM MgCl_2_ and 500 µM EGTA. To evaluate the Δψ*m* for each experimental condition, 2×10^7^ parasites were incubated with 10 µg/mL JC-1 during 40 min, with readings made every minute using a microplate reader (SpectraMax spectrofluorometer, Molecular Devices). The relative Δψ*m* value was obtained calculating the ratio between the reading at 590 nm and the reading at 530 nm (590∶530 ratio) – since mitochondrial de-energization leads to an accumulation of green fluorescence monomers, the decrease in the red/green fluorescence intensity ratio indicates a collapse in the mitochondrial transmembrane potential. Cells were also incubated in the presence of carbonyl cyanide 4-(trifluoromethoxy)phenylhydrazone (FCCP) at 1 µM, a mitochondrial protonophore, during the experiment as a positive control of the depolarization of the mitochondrial membrane. FCCP at the concentration of 2 µM was added at the end of all experiments to abolish Δψ*m*. This allowed comparison of the magnitude of Δψ*m* under the different experimental conditions.

### Ultrastructural analyses

Promastigote forms of *L. amazonensis* were cultured in Schneider's medium supplemented or not with the calpain inhibitor MDL28170 for 72 h in the absence (control) or the presence of the IC_50_ or two times the IC_50_ doses of MDL28170. For the observation of the ultrastructure modifications by transmission electron microscopy, promastigotes were fixed overnight at 4°C with 2.5% glutaraldehyde in 0.1 M cacodylate buffer, pH 7.2. After fixation, cells were washed in cacodylate buffer and post-fixed with 1% OsO_4_, 0.8% potassium ferrocyanide and 5 mM CaCl_2_ in the same buffer for 1 h at room temperature. Cells were then washed in the same buffer, dehydrated in graded acetone, and embedded in Epon. Ultrathin sections were mounted on 300-mesh grids, stained with uranyl acetate and lead citrate and observed using a FEI Morgagni F 268 microscope [19]. For scanning electron microscopy observation, cells over coverslips were fixed and post-fixed after 72 h of drug treatment as described above and dehydrated in graded series of acetone (30–100%). Cells were dried by the critical point method, mounted on stubs, coated with gold (20–30 nm) and observed in a Jeol JSM 6490LV scanning electron microscope [20].

### Surface binding of Annexin-V

The translocation of phosphatidylserine (PS) from the inner side to the outer layer of the plasma membrane is a common alteration during apoptosis in eukaryotic systems [21]. Annexin-V, a Ca^2+^-dependent phospholipid-binding protein with affinity for phosphatidylserine, is routinely used to label externalization of PS. Since Annexin-V can also label necrotic cells following the loss of membrane integrity, simultaneous addition of PI, which does not permeate cells with an intact plasma membrane, allows discrimination between apoptotic cells (Annexin-V-positive, PI-negative), late apoptotic/necrotic cells (both Annexin-V- and PI-positive) and live cells (both Annexin-V- and PI-negative). Double staining with Annexin-V and PI is also routinely used to measure the apoptotic effects of distinct drugs on the plasma membrane of *Leishmania* promastigotes [22]. In this sense, promastigote forms of *L. amazonensis* (5×10^6^ cells) were incubated or not with MDL28170 at either IC_50_ or two times the IC_50_ doses for 72 h. Cells were then centrifuged (4500×*g*, 5 min), washed twice in cold PBS, pH 7.2, and ressuspended in Annexin-V binding buffer (10 mM HEPES, 140 mM NaCl, 2.5 mM CaCl_2_) at pH 7.4. Annexin-V conjugated to Alexa-Fluor and PI were then added according to the manufacturers' instructions (Invitrogen, USA), and incubated for 20 min in the dark at 20–25°C. The intensity of labeling of Annexin-V conjugated to Alexa-Fluor was recorded in a FACSCalibur (BD Biosciences) flow cytometer and analyzed with Summit software, and the percentage of positive cells was assessed for each histogram. Miltefosine, an established inducer of apoptosis in *L. amazonensis* promastigotes (at 40 µM for 24 h) was used as a positive control [23], [24].

### Analysis of the cell cycle progression

The DNA fragmentation in apoptotic cells translates into a low fluorescence intensity (sub-G_0_/G_1_ phase) compared with cells in G_1_ phase of the cell cycle, and the intensity of staining with PI is correlated with the amount of DNA [25]. In order to determine the changes induced by MDL28170 on the cell cycle of *L. amazonensis*, parasites were treated or not with MDL28170 and incubated in the presence of PI prior to the analysis by flow cytometry. Promastigote forms of *L. amazonensis* (10^7^ cells) were incubated or not with MDL28170 at either IC_50_ or two times the IC_50_ doses of MDL28170 for 72 h and then washed twice with PBS, pH 7.2. Cells were fixed in chilled methanol and incubated overnight at −20°C. After two washes in PBS, the promastigotes were resuspended in 0.5 mL of PI (100 µg/mL in PBS) containing RNase A (248 U/mL), and the mixture was incubated for 20 min in the dark at room temperature. The fluorescence intensity of PI was analyzed with a FACSCalibur (BD Biosciences) flow cytometer and CellQuest software. A total of 10,000 events were acquired in the region previously established as that corresponding to the parasites. Miltefosine (at 40 µM for 24 h) served as the positive control [23], [24].

### *In situ* detection of DNA fragmentation of promastigotes

The detection of internucleosomal DNA cleavage is among the most characteristic and biochemical phenomena inherent in the process of apoptosis [26]. DNA fragmentation within the cell can be analyzed by Terminal Deoxynucleotidyltranferase (TdT)-mediated dUTP Nick End Labeling (TUNEL) using an APO-BrdU™ TUNEL Assay Kit according to the manufacturer's instructions. The TUNEL technique is able to quantify the proportion of DNA fragments by binding to BrdU via TdT. Thus, the amount of DNA fragments is directly proportional to the fluorescence obtained by BrdU incorporation and labeling by anti-BrdU antibody conjugated to Alexa Fluor 488 [27]. Briefly, promastigotes were incubated with IC_50_ or two times the IC_50_ doses of MDL28170 for 72 h, washed twice in PBS (pH 7.2) and fixed in 1% paraformaldehyde for 15 min. The cells were washed again in PBS and incubated in ice-cold 70% (v/v) ethanol for 30 min in a −20°C freezer. After washing for ethanol remotion, these cells were allowed to react with TdT enzyme in DNA-labeling solution for 60 min at 37°C. The samples were incubated with antibody staining solution containing the Alexa Fluor® 488 dye–labeled anti-BrdU antibody. Finally, the cells were resuspended in PBS before data acquisition using a FACSCalibur (BD Biosciences) flow cytometer and CellQuest software.

### DNA fragmentation assay by agarose gel electrophoresis

Qualitative analysis of fragmentation was performed by agarose gel electrophoresis of total genomic DNA extracted from untreated, miltefosine-treated promastigotes (positive control) and IC_50_ or two times the IC_50_ doses of MDL28170. Cell pellets consisting of 10^8^ promastigotes were lysed in lysis buffer (10 mM Tris-HCl, pH 8.5 mM EDTA, 0.5% SDS, 200 mM NaCl and 100 µg/mL pronase E), vortexed and incubated for 1 h at 37°C. Two volumes of absolute ethanol were added to the lysate and the samples were thoroughly mixed. These samples were centrifuged for 15 min at 14.000 rpm. The supernatants were discarded and the pellets were allowed to dry at room temperature. The genomic DNA was resuspended in 200 µL of 10 mM Tris-HCl, 0.1 mM EDTA (pH 7.5) and run on a 1.2% agarose gel containing ethidium bromide for 2 h at 85 V and visualized under UV light. A 100 pb DNA ladder (Invitrogen) was included as molecular size pattern.

### Statistical analysis

All experiments were performed in triplicate in three independent experimental sets. Data were analyzed statistically by means of one-way ANOVA using GraphPad Prism computer software.

## Results and Discussion

**Figure 1:**
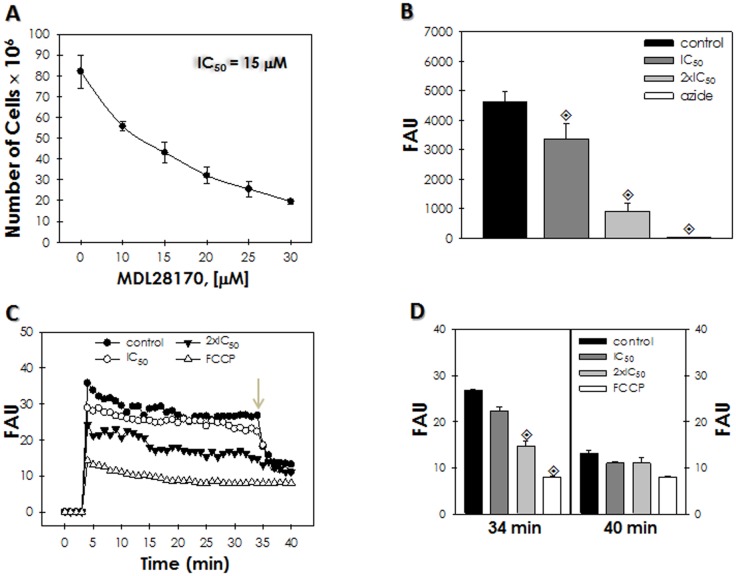
Effects of the calpain inhibitor MDL28170 on *Leishmania amazonensis* promastigotes growth, viability and mitochondrial function. (A) Parasites were treated with different concentrations of the inhibitor and growth was estimated after 72 h. The IC_50_ dose was calculated to be 15 µM. All values were significantly different from each other (*P*<0.0001). (B) Graphical representation of the level of resazurin reduction, expressed as fluorescence arbitrary units (FAU), in comparison to control, non-treated cells. Parasites were also treated with sodium azide to obtain non-viable cells. *P* values were obtained comparing the control cells with the treated groups, and the diamond symbol indicates that *P*<0.05. (C) Evaluation of Δψ*m* values (expressed as FAU) after the addition of the JC-1 fluorochrome. The arrow indicates the time point (34 min) at which the uncoupler FCCP was added at 2 µM to collapse mitochondrial potential. (D) Comparison of the Δψ*m* values (expressed as FAU) shown in (C) before (34 min) and after (40 min) the addition of the uncoupler FCCP.

Initially, the calpain inhibitor MDL28170 was added to *L. amazonensis* promastigote forms in different concentrations, and the cellular growth was compared with a control culture after 72 h. Our results showed that MDL28170 arrested the growth in a dose-dependent manner, and the IC_50_ after 72 h was calculated to be 15 µM (Figure 1A). In parallel, DMSO, the solvent of this drug, did not affect the parasite growth behavior (data not shown).

Parasite viability was then determined using the resazurin assay, which showed a great decrease in resazurin reduction after MDL28170 treatment, mainly with the two times the IC_50_ dose of the drug, when compared to non-treated control cells (Figure 1B). This result indicated that the calpain inhibitor induced loss of parasite viability in a concentration-dependent manner. Incubation with JC-1 showed that cells treated with the calpain inhibitor at two times the IC_50_ dose had a significant reduction of Δψ*m* (Figure 1C,D) when compared with the control (untreated) parasites. In addition, pre-incubation with FCCP resulted in decreased mitochondrial staining with JC-1 (Figure 1C). After 34 min of JC-1 uptake, the addition of 2 µM FCCP fully collapsed the Δψ*m*, including control parasites (Figure 1C,D). Since loss of mitochondrial membrane potential was mainly observed in cells treated with two times the IC_50_ dose, this may be considered a secondary effect of MDL28170 treatment.

**Figure 2:**
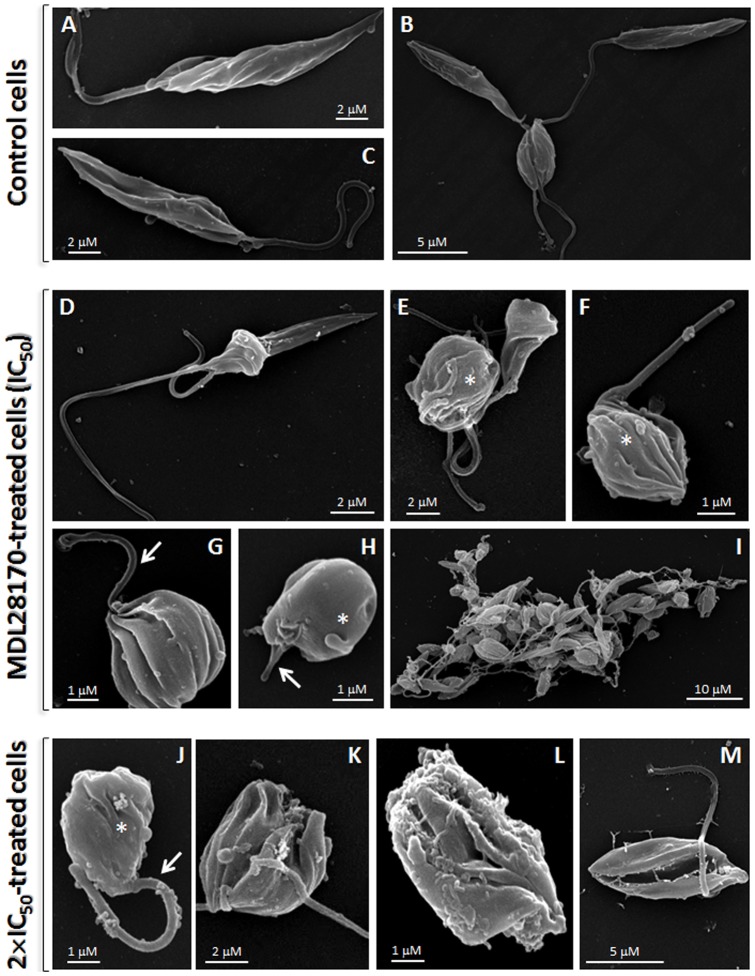
Scanning electron microscopy of *Leishmania amazonensis* promastigotes after treatment with MDL28170. In untreated, control promastigotes (A–C), note the elongated body and emerging flagellum. (D–I) Treatment with the IC_50_ dose (D–I) and two times the IC_50_ (2×IC_50_) dose (J–M) of MDL28170 for 72 h caused shortening of the parasite body (E–H,J) and of the flagellum (arrows), membrane protrusions and ruffling of the plasma membrane (asterisks), appearance of cells with bizarre shape (D,K–M) and aggregation of cells (I).

Having observed that MDL28170 treatment induced loss of parasite viability as well as depolarization of mitochondrial membrane (Figure 1), we decided to investigate the effects of this calpain inhibitor on the ultrastructure of MDL28170-treated parasites. In scanning electron microscopy, the control, non-treated parasites retained their normal features, like a stable cell surface, the typical elongated shape and long flagellum at all-time points (Figure 2A–C). Promastigotes treated with the IC_50_ and two times the IC_50_ concentrations of MDL28170 showed different degrees of morphological changes. Many cells exhibited shrinking/rounding up responses and also the loss of flagellum, consistent with the decrease in their viability (Figure 2E–H and 2J). In addition, some cells were bizarrely shaped (Figure 2D, E, K–M), sometimes presenting membrane protrusions resembling surface blebs as well as ruffling of the plasma membrane (Figure 2E, F, H, J–M). This type of morphological effect has been previously shown for apoptotic-like death induced by distinct compounds [28]. When promastigotes were incubated with the IC_50_ dose, aggregation of cells was visible. Some of the cells that were clumped together presented morphological alterations and cell debris (Figure 2I).

**Figure 3:**
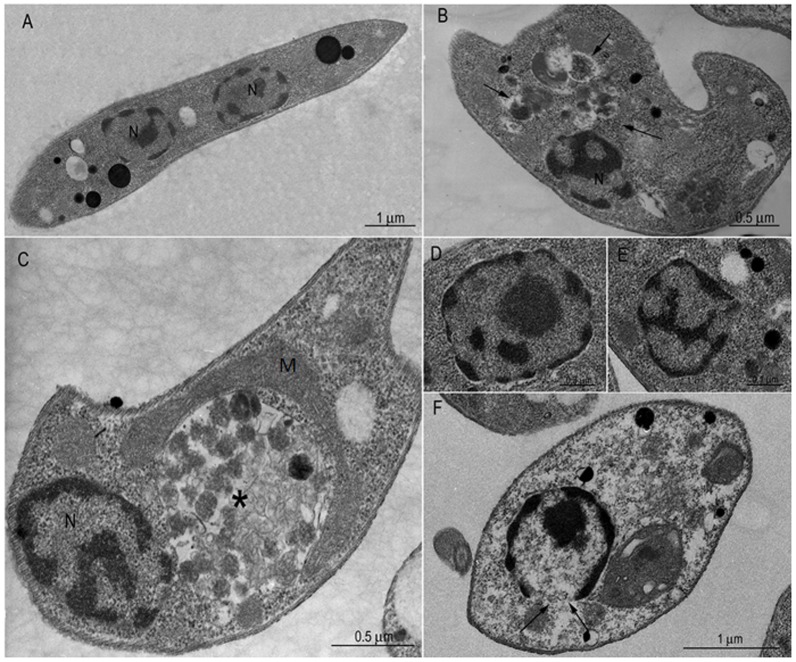
Transmission electron microscopy of *Leishmania amazonensis* promastigotes after treatment with MDL28170. (A) Control, untreated cell: note the elongated body and the normal aspect of the intracellular organelles (N, nucleus). In MDL28170-treated promastigotes both at IC_50_ (B–C) and two times the IC_50_ (2×IC_50_) doses (D–E), the ultrastructural changes show intense vacuolization in the cytoplasm (arrows in B), disorganization of compartments of the endocytic pathway containing multivesicular bodies-like vesicles (C, asterisk), altered chromatin condensation pattern (D–F) and apparent loss of nuclear integrity (F, small arrows). M, mitochondrion.

In transmission electron microscopy, the results showed ultrastructural changes after 72 h of incubation with the inhibitor at IC_50_ (Figure 3B–C) and two times the IC_50_ doses (Figure 3D–F) in comparison to non-treated parasites (Figure 3A). MDL28170-treated cells showed an intense vacuolization in the cytoplasm (Figure 3B–C). In addition, the calpain inhibitor was also able to induce an intense disorganization of the endocytic pathway, which is distended and presented reduced electron density as well as the accumulation of small vesicles characteristic of the multivesicular body network (Figure 3B–C). The mitochondrion displayed a normal shape and density when cells were incubated with the calpain inhibitor at IC_50_ dose (Figure 3C), confirming the secondary effect of MDL28170 on this organelle, as previously shown in the estimation of Δψm (Figure 1C). Some parasites incubated with two times the IC_50_ dose presented an empty cytoplasm and nucleoplasm and an apparent rupture of the nuclear envelope (Figure 3F). Besides these ultrastructural changes, the detection of an altered chromatin condensation pattern (Figure 3D–E) in *L. amazonensis* promastigotes during the MDL28170 treatment is suggestive of an apoptosis-like cell death process, as described elsewhere in trypanosomatids treated with other drugs [28].

**Figure 4:**
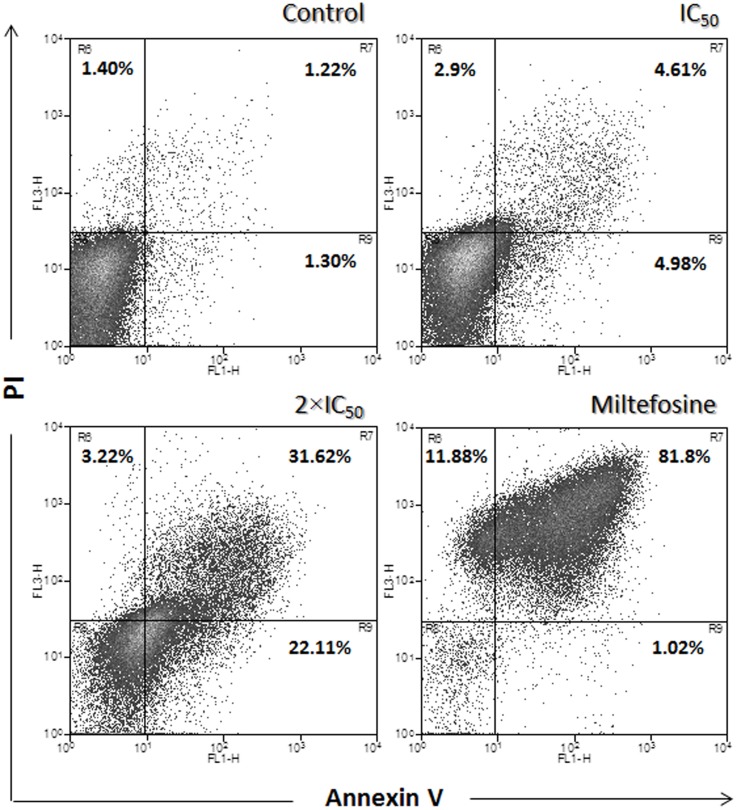
Surface binding of Annexin-V in MDL28170-treated *Leishmania amazonensis* promastigotes. Late exponential-phase promastigotes of *Leishmania amazonensis* were treated or not with MDL28170 (IC_50_ and two times the IC_50_ [2×IC_50_] doses) for 72 h, co-stained with PI and annexin V–Alexa Fluor 488 and analyzed by flow cytometry. Cells treated with miltefosine (40 µM for 24 h) were used as a positive control.

Based on the data suggesting nuclear condensation in promastigotes, we sought to further investigate the possibility of apoptotic-like cell death mediated by the calpain inhibitor. In order to do so, promastigotes treated with MDL28170 (IC_50_ and two times the IC_50_ doses) for 72 h were double-stained with Annexin-V-FITC and PI. The percentage of promastigotes that were positive only for Annexin-V was 4.98% after treatment with the IC_50_ dose of MDL28170 and 22.11% when cells were treated with two times the IC_50_ (Figure 4), which suggests that these cells were in the early stages of apoptosis-like death. The number of cells that were both Annexin-V- and PI-positive (Figure 4) was 4.61% and 31.62%, respectively, which is related to late events of apoptosis-like and/or necrosis. No significant difference in the number of apoptotic-like and necrotic cells was observed after 96-h incubation (data not shown). Non-treated cells were Annexin-V- and PI-negative (Figure 4), and the same result was seen in cells treated only with DMSO, the vehicle of drug (data not shown), which confirms the viability of cells in these conditions. Alternatively, promastigotes treated with miltefosine were used as a positive control in this experiment [23], [24]. After 24 h of incubation with miltefosine at 40 µM, 1.02% of cells were apoptotic-like (Annexin-V-positive and PI-negative) and 81.8% of cells were already in late apoptotic-like phase or necrosis (Annexin-V-positive and PI-positive) (Figure 4). The observation of most of cells being Annexin-V-positive and PI-positive on miltefosine treatment beyond 24 h was already demonstrated to be associated to cells at a very advanced stage of apoptosis-like death and resemble necrotic cells, which are difficult to discriminate [29].

A critical point in the analysis of the results presented in Figure 4 is concerned to the presence or lack of PS in *Leishmania* spp. cell membrane. In eukaryotic cells, PS is restricted to the inner plasma membrane leaflet, and any change in this distribution usually triggers a physiological event such as the clearance of apoptotic cells [21]. PS exposure has also been implicated as an apoptotic marker [22] as well as in the infectivity of *Leishmania:* in this sense, Wanderley et al. [30] showed that apoptotic, PS-positive *L. amazonensis* promastigotes are present in the sand fly gut, being part of the infective inoculums in natural infections, and that the apoptotic sub-population inhibited host macrophage inflammatory response. However, there are conflicting results regarding the presence of PS in promastigote membrane fractions [31], [32]. In this regard, Weingartner et al. [31] highlighted that PS exposure on the cell surface of promastigote forms has been detected with either anti-PS antibodies or Annexin-V, both of which able to bind to other phospholipids present in the cell membrane. The same group performed a combined lipid analysis of *Leishmania* promastigotes and no evidence was found for the presence or synthesis of PS. In addition, binding of Annexin-V upon permeabilization or miltefosine treatment was determined not to be restricted to PS but included several other phospholipids such as phosphatidylehtanolamine, phosphatidylinositol and cardiolipin, being suggested that binding of Annexin-V to the cell surface of promastigote forms may likely to be a consequence of changes in the transbilayer plasma membrane lipid arrangement. Contrary to this argument, Imbert et al. [32] found out in a lipidomic study focused on *L. donovani* that PS can be considered as a detectable, although a minor, component of membrane phospholipids, and increasing amounts were found in miltefosine-treated promastigotes. As pointed out by Weingartner et al. [31], a potential reason for this discrepancy could be that in late logarithmic phase, as performed in the present work, promastigotes do synthesize PS, allowing Annexin-V binding. It is also possible that *Leishmania* amastigotes synthesize PS and regulate its transbilayer distribuition: in an infection with *L. amazonensis*, amastigote forms may display PS on their external membrane outer leaflet to ensure leishmanial intracellular survival due to host phagocyte inactivation, which has been named apoptotic mimicry and it is modulated by the host [33].

**Figure 5:**
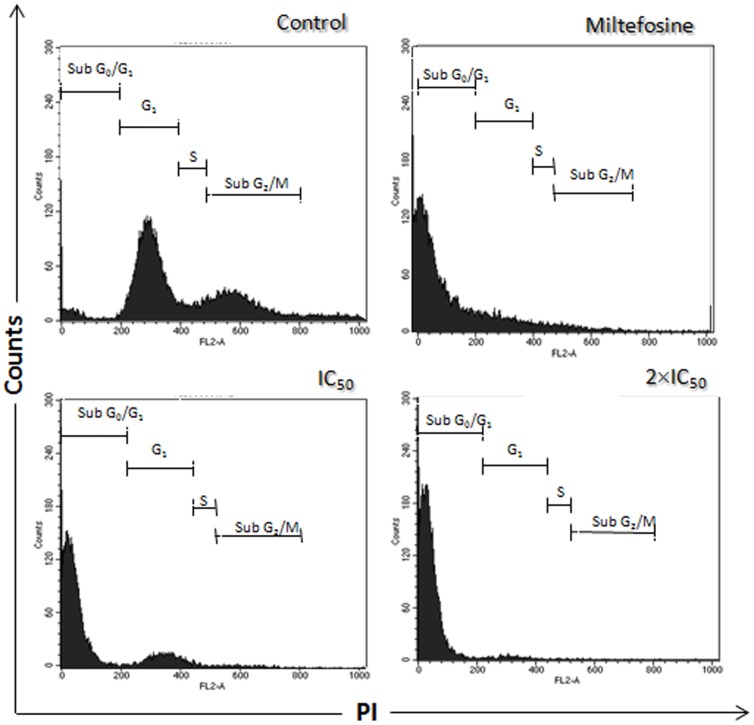
Increase in the sub G_0_/G_1_ DNA-containing *Leishmania amazonensis* promastigotes population after MDL28170 treatment. The DNA content degradation profiles of exponential-phase promastigotes of *Leishmania amazonensis* treated or not with MDL28170 (IC_50_ and two times the IC_50_ [2×IC_50_] doses) were assessed by flow cytometry after cell permeabilization and PI staining. DNA fragmentation was quantified by measuring the cell population in the sub G_0_/G_1_ DNA region. Cells treated with miltefosine (40 µM for 24 h) were used as control.

Besides Annexin-V binding in the cell surface, the treatment of promastigotes of *L. amazonensis* with the calpain inhibitor at IC_50_ and two times the IC_50_ doses by 72 h was able to induce drastic changes in the cell cycle of the parasites (Figure 5). The inhibitor induced a significant increase in the proportion of cells in the sub-G_0_/G_1_ phase in a concentration-dependent manner, with consequent decrease of cells in G_0_/G_1_, S (synthesis) and G_2_/M phases when compared either to control cells or cells exposed only to DMSO (Figure 5). Miltefosine, used as a positive control in these experiments, is able to induce cell-cycle arrest, presenting a large number of cells in sub-G_0_/G_1_ phase (Figure 5). These results are also in accordance with the hypothesis that MDL28170 inhibited leishmanial viability through the apoptosis-like pathway.

**Figure 6:**
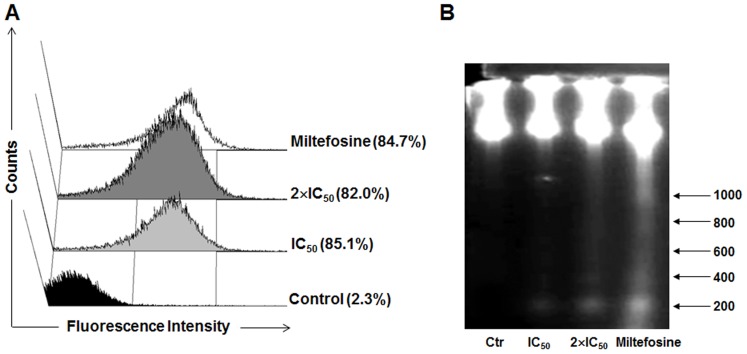
Analysis of DNA fragmentation in MDL28170-treated *Leishmania amazonensis* promastigotes. Cells were incubated or not (Control, Ctr) with IC_50_ or two times the IC_50_ (2×IC_50_) doses of MDL28170 for 72 h. In the analysis of TUNEL positivity (A), cells were fixed, stained with PI and dUTP-FITC in the presence of terminal deoxynucleotidyl transferase and RNase enzyme and analyzed by flow cytometry. The DNA profiles of total genomic DNA by 1.2% agarose gel electrophoresis (85 V for 2 h) are shown in B. DNA from promastigotes treated with 40 µM miltefosine for 24 h were used as a positive control of oligonucleossomal fragmentation. The DNA fragments sizes, based on 100 bp DNA ladder migration, are expressed in bp.

The cell cycle profile shown in Figure 5 may be correlated to late events of apoptosis-like death, such as internucleosomal DNA fragmentation [27]. Therefore, the TUNEL assay was performed in order to verify whether the calpain inhibitor was able to induce DNA fragmentation in promastigotes of *L. amazonensis*. The treatment of *L. amazonensis* promastigotes with MDL28170 for 72 h at the concentrations of IC_50_ and two times the IC_50_ doses induced genomic DNA fragmentation. This profile can be seen in Figure 6A, through the incorporation of BrdU and the increase in the number of cells stained by anti-BrdU, compared to non-treated promastigotes, which did not show any TUNEL-positive cells. The promastigotes treated with 40 µM miltefosine also showed an intense staining by anti-BrdU, as represented in Figure 6A. DMSO was not able to induce DNA fragmentation (data not shown).

The pattern of DNA fragmentation was assessed by electrophoresis on agarose gels using the total DNA extracted from promastigotes treated or not with the calpain inhibitor. In cells treated with MDL28170 at IC_50_, it was not possible to observe a typical oligonucleossomal DNA fragmentation (Figure 6B). This result may indicate that TUNEL assay was more sensitive than agarose gel electrophoresis in monitoring DNA cleavage following exposure to MDL28170; alternatively, DNA fragmentation following MDL28170 treatment may be a secondary effect, as previously observed in the mitochondrion (Figure 1C,D). However, in cells treated with MDL28170 at two times the IC_50_ dose and miltefosine at 40 µM (Figure 6B), we observed DNA fragmentation in multiples of 200 base pairs, a characteristic of apoptosis-like death, and a certain degree of smearing in DNA, mainly after miltefosine treatment, which may be correlated to a very advanced stage of apoptosis-like and/or necrotic cells, as previously observed in PI-positive cells after Annexin-V staining (Figure 4). No fragmentation of the genetic material was observed in untreated cells, used as a control (Figure 6B), and DMSO was not able to induce DNA fragmentation (data not shown).

Apoptosis is originally considered a characteristic of multicellular organisms, and it is defined as a form of programmed cell death in which a sequence of controlled events leads to locally and temporally defined self-destruction without inducing or evoking inflammatory responses, in contrast to necrosis, which is a form of cell death that results from acute tissue injury and provokes an inflammatory response. Recent data suggest that a mechanism with many similarities to metazoan apoptosis is operative in unicellular eukaryotes, including trypanosomatids [34], [35]. In these species, apoptosis is suggested to be useful in the establishment of infection in several ways, including the control of parasite numbers in response to limited resources, within the insect vector or mammalian host, which would provide a means for the perpetuation of infection. In such a way, the coupling of cell-cycle control and proliferation with this programmed form of cell death would provide a means for the selection of the fittest cells in hostile environments [35]. In *Leishmania* spp., it has also been suggested that a regulated cell death may be useful to trypanosomatids for the avoidance of an inflammatory response and for the silencing of the immune system, allowing in this way the intracellular survival of non-apoptotic parasites [36].

Some of the morphological features found in apoptotic multicellular lineages are also detected in trypanosomatids, such as cell rounding up, reduction of the cellular volume, membrane blebbing, chromatin condensation and engulfment by host phagocytes, dissipation of the mitochondrial membrane potential, Annexin-V binding to the outer leaflet of the plasma membrane, cytochrome c release, maintenance of an intact plasma membrane until late stages of the process, induction of proteases and DNA cleavage [34]. However, the question of whether the expression of apoptotic markers is a reliable readout for parasite cell death was recently raised [37], [38]. The controversial discussion of the existence of programmed cell death in *Leishmania* has been discussed by Foucher et al. [37], due to the staurosporine-induced expression of several apoptotic markers in *L. donovani* promastigotes in the absence of cell death. In addition, Proto et al. [38] proposed that cell death in parasitic protozoa may be classified into just two primary types, necrosis or incidental death, due to the absence of key executioners found in higher eukaryotes for the initiation and execution of regulated cell death. In the incidental death, harsh non-physiological conditions, like drug treatment, lead to phenotypes with inconsistent or overlapping features of mammalian apoptosis that are apparently typical of multiple death subtypes. In this sense, although these discrepancies do not entirely preclude functional regulated cell death mechanisms in protozoa, they must differ from the processes in higher eukaryotes and the dedicated molecular machinery has yet to be properly identified [38].

In mammalian cells, the main executors of apoptosis after mitochondrial membrane potential dissipation are nucleases and the cysteine peptidases called caspases [39], but pathways of caspase-independent apoptosis are found, with central players peptidases being cathepsins, calpains, granzymes A and B and the peptidases of the proteasome [40]. Conflicting roles for calpain activity in contributing to the promotion and/or suppression of apoptosis have been proposed in mammals, being suggested that calpains must have a wide influence over many apoptotic processes, and their specific roles during apoptosis may differ depending on cell type and the nature of the apoptotic stimulus [9]. Trypanosomatids do not have caspase genes, and therefore they should undergo a caspase-independent apoptosis-like cell death, which involves putatively proteasomal peptidases and metacaspases (cysteine peptidases with similar folds as caspases), but caspase-like activity was already assessed by the cleavage of caspase-specific substrates induced by the anti-leishmanial drugs Pentostam and amphotericin B and the inhibitory effect of caspase-specific inhibitory peptides [34], [41]. It is proposed that peptidases with little homology, but with overlapping activity to metazoan caspases, may be involved in the execution of apoptosis-like cell death in trypanosomatids, such as the leishmanial cathepsin L-like cysteine peptidases CPA/CPB [42] and the cathepsin B-like cysteine peptidase CPC [43]. In this sense, the sequential involvement of both cathepsin and calpain family could represent a prototype of the caspase cascade occurring in trypanosomatids apoptosis-like death [35].

The results presented in the current work raised the question as to whether calpains should be the main target of MDL28170 against the parasite. This compound is considered a relatively specific calpain inhibitor, but it cannot be ruled out that this inhibitor acts on other cysteine peptidases to a lesser extent, mainly cathepsins B [44], and as such it remains possible that a cysteine peptidase other than calpain, such as the previously mentioned CPC, is mechanistically involved in the expression of apoptotic markers detected in the parasite. Interestingly, Paris et al. [23] explored the possibility of peptidases activity being involved in the induction of apoptosis-like death by miltefosine. Pretreatment of *L. donovani* promastigotes with two broad caspase inhibitors, as well as a broad peptidase inhibitor, calpain inhibitor I, prior to miltefosine exposure reduced the percentage of cells present in the G_1_ peak region but did not prevent cell shrinkage or Annexin-V binding, which is suggestive that at least part of the apoptotic-like machinery operating in promastigotes involves peptidases. Calpain inhibitor I, which inhibits calpains, cathepsins B and L, as well as the proteasome, was the most efficient compound in interfering with DNA fragmentation, which was also confirmed by the marked inhibition of the nitric oxide-induced [45] and antimonial-induced [46] oligonucleosomal DNA fragmentation of *Leishmania* spp. amastigotes. As pointed by the authors, these data further imply that peptidases are most likely involved in the molecular signaling leading to nuclear changes but the broad spectrum of activity of these inhibitors precludes any clear identification of their nature.

The expression of apoptotic markers in trypanosomatids is triggered in response to diverse stimuli such as heat shock, reactive oxygen species, prostaglandins, starvation, antimicrobial peptides, antibodies, serum as a source of complement, mutations in cell cycle regulated genes, as well as by antiparasitic drugs [34]. Among the latter, racemoside A [27], miltefosine [23], [24], antimonials [46], pentamidine [47] and nelfinavir [48] induce the expression of apoptotic markers in *Leishmania* spp., among others. Interestingly, a distinct pathway leading to apoptosis-like death was found upon exposure of trypanosomatids to microtubule interfering agents, such as taxol and certain alkaloids [49], possibly by disruption of microtubule networks within the mitochondrion [50] or via the direct opening of the permeability transition pore [51]. The knocking down of centrin in *L. donovani* amastigotes, a cytoskeletal calcium-binding protein that regulates cytokinesis in trypanosomatids, induces apoptotic-like death [52]. In this sense, a large body of evidence suggests that cytoskeleton participates in morphological changes during apoptotic progression induced in different mammalian cell death models, and calpains are known to recognize and cleave cytoskeleton proteins [53].

These data may be correlated to the expression of calpain-like proteins (CALPs) in trypanosomatids, since a cytoskeleton-associated protein, named CAP5.5, which was found in *Trypanosoma brucei* procyclic forms, is characterized by the similarity to the catalytic region of calpain-type peptidases [54]. CAP5.5 was detected evenly distributed across the subpellicular microtubule corset, and although there is an overall similarity with the catalytically active domain of calpains, only one of the three amino acids constituting the active site in classical calpains (CHN) is conserved in CAP5.5 (SYN) [54]. Through RNAi experiments that selectively targeted either *CAP5.5* or its paralog, named *CAP5.5V*, Olego-Fernandez et al. [55] subsequently showed that *CAP5.5* is essential for cell morphogenesis in procyclic forms, while *CAP5.5V* is expressed and essential in bloodstream forms. In addition, it was demonstrated that the paralogous genes provide analogous roles in cytoskeletal remodeling for the two life cycle stages, being necessary for the correct morphogenetic patterning during the cell division cycle and for the organization of the subpellicular microtubule corset. The authors suggested that loss of proteolytic activity may have been an important step in the functional evolution of these CALPs, mainly to act as microtubule-stabilizing proteins.

As previously detected in *T. brucei*, CALPs were also found as microtubule-interacting proteins in *T. cruzi*. In the latter, the H49 antigen, which encodes a high molecular mass repetitive protein composed of 68-aminoacid repeats tandemly arranged, is located in the cytoskeleton of epimastigote forms, mainly in the flagellar attachment zone, and sequence analysis demonstrated that the 68-aa repeats are located in the central domain of CALPs. Critical alterations in the catalytic motif suggest that H49 protein lack proteolytic activity. The so-called H49/calpains could have a protective role, possibly ensuring that the cell body remains attached to the flagellum by connecting the subpellicular microtubule array to it [56]. Inexact H49 repeats were found in the genomes of other trypanosomatids, including *T. brucei, L. major, L. infantum* and *L. braziliensis*, with less than 60% identity to H49 and located in CALPs, including *T. brucei* CAP5.5 [56]. These data should be further explored in order to correlate the presence of cytoskeleton-associated CALPs and the cell cycle in trypanosomatids.

With the establishment of the leishmanicidal activity of MDL28170 [7], it became important to study the mechanism of action of this calpain inhibitor. Through the use of combined techniques, such as resazurin assay, JC-1 staining, electron microscopy, Annexin-V labeling, TUNEL assay and agarose gel electrophoresis, the data presented in the current study support the hypothesis that MDL28170 induces the activation of classical markers of apoptosis in promastigotes of *L. amazonensis*. Altogether, the results described in the present work not only provide a rationale for further exploration of the mechanism of action of calpain inhibitors against trypanosomatids, but may also widen the investigation of the potential clinical utility of calpain inhibitors in the chemotherapy of leishmaniases. In this sense, preliminary results showed no deleterious effects on mouse peritoneal macrophages viability when MDL28170 was applied at IC_50_ and two times the IC_50_ values determined for *L. amazonensis* promastigotes, and a significant reduction in the association index of *L. amazonensis* with macrophage cells was found during the *in vitro* treatment with the calpain inhibitor at both concentrations for 24 h. In addition, the IC_50_ value of MDL28170 for axenic amastigotes was significantly lower than the value determined for promastigotes (data not shown). Further studies will probably delineate the exact roles of trypanosomatid peptidases as possible executors of apoptosis-like cell death in these microorganisms.
